# The Impact of Atmospheric Parameters on the Dielectric Permittivity Values of SikaBlock^®^-M150 and Other Rigid Polyurethane Foams Measured with a Capacitive One-Side Access Sensor

**DOI:** 10.3390/s22207859

**Published:** 2022-10-16

**Authors:** Ilze Beverte, Sergejs Gaidukovs, Janis Andersons, Vilis Skruls

**Affiliations:** 1 Institute for Mechanics of Materials, University of Latvia, LV-1004 Riga, Latvia; 2 Institute of Polymer Materials, Faculty of Material Science and Applied Chemistry, Riga Technical University, LV-1048 Riga, Latvia

**Keywords:** polyurethane foams, capacitive sensor, one-side access, dielectric permittivity, measurements, relative humidity, temperature, absorption, warm-up drift

## Abstract

A shortage of research on the impact of atmospheric parameters on the measured dielectric permittivity values of rigid polyurethane (PU) foams was identified. Therefore, the impact of temperature, pressure, and relative humidity of air in the test room on the measured values of dielectric permittivity of rigid PU foams of different densities as well as monolithic polyurethane was investigated in a year-long experimental research study with a capacitive one-side access sensor. It was shown that relative humidity has the highest correlation with the dielectric permittivity values of rigid PU materials. The detected values of parameters were linked to the water vapour mass in ambient air and its correlation with permittivity of the investigated materials was determined. The warm-up drift and warm-up time of the spectrometer were estimated experimentally. A novel methodology was demonstrated to determine the true permittivity spectrum of rigid PU foams without any involvement of the environmental chamber, desiccators, or saturated salt/water solutions. A relative increase in the measured dielectric permittivity value was estimated numerically for the entire density range of rigid PU foams, i.e., 33–1280 kg/m^3^ (including monolithic PU).

## 1. Introduction

Applications of rigid polyurethane (PU) foams of densities in the range of 130–170 kg/m^3^ include substructures for design, styling and clay models, the manufacturing of styling models and design studies, test milling, etc., in automobile, machine building, and structural industries [1,2,3,4,5]. The foams are also used as encapsulants for electronic components to mitigate harsh thermal and mechanical environments, as well as to provide electrical insulation [6]. The foams exhibit a low dielectric interference and their dielectric permittivity (permittivity) is nearly non-dispersive; therefore, a good dielectric performance can be ensured in a wide frequency range [7,8,9]. SikaBlock^®^-M150 PU foams are dense, easily workable, and industrially manufactured (Sika JSC, Baar, Switzerland) with a fine structure, exhibiting a low dust formation when milled. The foams have a low thermal conductivity coefficient and very low linear thermal expansion coefficient [4,5]. As a rule, rigid PU foams have ≥ 98% closed cells [1,2,3].

Polyurethanes make up a group of generally polar polymers with a surface free energy ≈40 mJ/m^2^ [2,3]. The dependence of rigid PU foams’ dielectric permittivity on the frequency of the applied electric field (i.e. the dielectric dispersion) was characterised by a dropping factor F in [7]. It was shown that for rigid closed-cell PUR foams in the considered low-frequency range of 10 Hz–0.33 MHz, even at densities 400 kg/m^3^–500 kg/m^3^, the F values do not exceed 5.0–5.5% and the dropping factor is F ≤ 1.2% for SikaBlock^®^-M150 PU foams.

As a non-metallic composite material, widely applied in industry, rigid PU foams require efficient non-destructive evaluation (NDE) methods for controlling the quality of manufactured items and materials. In the NDE of non-metallic materials, in the frequency band up to 10 MHz, one of the main testing methods is the capacitance method [7,9]. In a direct way, it helps to determine the real and imaginary part of the complex dielectric permittivity, as well as their dependence on frequency, temperature, relative humidity, relaxation parameters, etc. [10]. Knowledge on the permittivity of rigid PU foams is necessary in NDE, in practical applications, related to the electromagnetic field, as well as air- and space-craft applications, etc. [7,8]. The method had wider application with developing capacitive one-side access (COSA) sensors [11,12,13,14,15]. They allow NDE by applying the sensor to one side of the testing object, without making test samples. COSA sensors are widely used in non-destructive testing, proximity/displacement measurements, material characterization, non-metallic material thickness determination, intelligent human interfacing, security systems, etc. [7,8,12,15].

The essential characteristics of a capacitive sensor’s stability are sensitivity, warm-up drift, and offset voltage [16,17,18,19]. The warm-up drift characterises the change in values of permittivity, measured by a capacitive sensor that results from the initial warming and thermal expansion of the components over a certain period [19]. An important parameter of capacitive sensors is the warm-up time to reach stabile and accurate readings. Allowing a capacitive sensor to warm up gives it time to reach thermal equilibrium and stabilize the measurement values.

The major technical difficulties, connected to the PU foams themselves, lie in low permittivity values. At excitation frequencies of 10 Hz–0.33 MHz, the permittivity of (a) SikaBlock^®^-M150 PU foams is measured as 1.23–1.27 and the permittivity of (b) rigid PU foams (with a density of 33 kg/m^3^) is measured as 1.050–1.048; i.e., only ~5% higher than the permittivity of vacuum [7,8].

COSA sensors can operate in indoor as well as outdoor environments [7,8,20,21,22]. Air temperature, atmospheric (barometric) pressure, and humidity are among the main atmospheric parameters. The dry air of the Earth’s atmosphere is a mixture of gases that contains ~78% nitrogen; ~21% oxygen; ~1% argon; <1% carbon dioxide; and traces of hydrogen, helium, and other “noble” gases (by volume). A varying amount of water vapor is present, ~1% on average (at sea level). At standard reference conditions [ISO 5011] T = 20 °C, p = 1013.25 hPa, and RH = 50%, the mass of water vapor in air is 8.05 g/m^3^. The capacity of air to contain water vapor increases as its temperature rises [23]. Relative humidity depends on air temperature and moisture content. Changes in temperature and humidity of the surrounding atmosphere can affect any capacitive structure and are particularly critical for capacitive sensors. Capacitive sensors are constructed with two or more conducting electrodes and an insulating support; the components change size in response to environmental factors, and long-term stability may be a concern [24,25].

The atmospheric moisture is absorbed by hygroscopic plastic materials, such as nylon, acrylic, PET, PBT, polyurethane, polycarbonate, etc. Monolithic PU attracts and holds water molecules via absorption from the surrounding environment. In [25], the impact of a varying weight percentage of a triethylenediamine catalyst and curing for a shorter time at a higher temperature on the mechanical properties, morphology, thermal stability, and water absorption of monolithic rigid polyurethane was investigated. It showed that a bigger amount of a catalyst and a higher curing temperature produce a more compact structure. A network of cross-links prevents the diffusion of water molecules and thus prevents them from residing in the available volume.

In plastic foams (a composite of “Polymer-gas”), the atmospheric moisture can be absorbed both into the open cells and into the polymeric elements. Due to the dominantly closed-cell structure, rigid PU foams have comparatively little propensity to absorb moisture or water in immersion tests [1,2,3], e.g., for PU foams of densities < 60 kg/m^3^, water absorption in small-scale tests is <2–3 vol.%/7days (ISO 2896:2001).

PU-based cellular material is considered to be a promising candidate for internal insulating the medium of composite post insulators of ultra-high voltage direct current transmission lines. While a lot of papers deal with the impact of humidity and temperature on electric breakdown strength, only single papers consider the same for dielectric permittivity. A series of rigid PU foams with various diphenylmethane diisocyanate contents were synthesized [26]. The PU foams specimens were put into a controlled temperature chamber. The effects of temperature and exposure time on the hygroscopicity of rigid polyurethane foams at various relative humidity levels 35%, 55%, 75%, and 100% were explored. An increase in absorbed moisture was identified with an increase in relative humidity and temperature. A saturation state in moisture absorption was detected. It was shown that the dielectric permittivity and dielectric losses increased significantly after moisture absorption. At the same time, research is limited to light-weight PU foams and artificially modelled humidity conditions. A search in scientific information sources reveals a shortage in data on the impact of natural atmospheric moisture on the dielectric permittivity of rigid PU foams, measured either in laboratory or outdoor conditions. A systematic investigation for rigid PU foams of different densities is lacking as well.

Polytetrafluoroethylene (PTFE, brand name Teflon) is a plastic material, known for lacking any dispersion of dielectric permittivity in a wide range of frequencies due to non-polar molecules and exhibiting only elastic polarization [8]. The well-known and stabile dielectric properties make PTFE an appropriate reference material for testing methodologies. Permittivity of PTFE is reported in [8] as ε′ = 2.1 at 60 Hz – 1 MHz and dissipation factor as tgδ = 0.00007 at 1 MHz. At 1 MHz the same properties are reported as ε′ = 2.2 and tgδ < 0.00012. PTFE is hydrophobic due to the chemical composition and geometrical structure of the surface; its surface free energy of PTFE is only ≈19 mJ/m^2^.

The given research aims to investigate the impact of the main atmospheric parameters—temperature, pressure, and relative humidity—on the measured dielectric permittivity values of rigid PU foams of different densities as well as of monolithic polyurethane, based on the data of a long-term experimental investigation with a capacitive one-side access sensor. The detected atmospheric parameters were linked to the water vapour mass in air. The warm-up drift and warm-up time of the spectrometer to reach stabile readings were estimated experimentally. Using the acquired data, a novel methodology was elaborated and demonstrated using the SikaBlock^®^-M150 rigid PU foams to calculate the true permittivity spectrum without any involvement of environmental chambers, desiccators, or salt/water solutions. A relative increase in the measured dielectric permittivity value was estimated numerically for the entire density range of rigid PU foams 33–1280 kg/m^3^, including monolithic polyurethane.

## 2. Materials and Methods

### 2.1. The Test Sample

The intensity maximum of a dielectric spectrometer’s low-frequency excitation field is located in a direct vicinity to the active area of a COSA sensor’s electrodes [7,8,10]. The penetration depth of the excitation field is a crucial characteristic for the sensor in determining the appropriate thickness of the test sample, depending on the parameters of the sensor and dielectric material of the sample. In the investigation, the penetration depth of the electric field is defined as the thickness h_3%_ of a sample, at which the measured value of electric susceptibility χ = ε − 1.0 is 3% less than the true value of electric susceptibility χ_t_ of an infinitely thick sample [8,15].

For PU foams of densities 50–228 kg/m^3^ and ε = 1.14 − 1.42 (f = 1 kHz), the penetration depth was estimated as 5.72 mm ≤ h ≤ 5.87 mm ± 0.02 mm [8]. The PU foams test sample has to be thick enough to provide the true value of permittivity. The lateral dimension (thickness h, parallel to the foams’ rise direction), around double the size of the penetration depth, was taken as appropriate for a PU foams sample of density ≈150 kg/m^3^: h≈13 mm. To evaluate the impact of the test sample’s transversal dimensions on the measured value of permittivity ε, the spectra were measured for two parallelepiped-shaped samples, (a) 13 × 65 × 65 mm and (b) 13 × 45 × 45 mm, as well as for (c) a cylindrical sample of h = 13 mm and diameter 43 mm, precisely matching the sensor’s active area of the same diameter. In the limits of measurements’ uncertainties, the measured permittivity spectra were the same for the three kinds of samples. The parallelepiped-shaped test sample of dimensions 13 × 65 × 65 mm was used in the further investigation. To minimise processing-caused slopes on the bottom surface, forming air gaps upon contact with the active area of the sensor [8], the sample was cut from the central part of a parallelepiped sample of the same height, but having around three times larger transversal dimensions.

### 2.2. Other Materials

The dielectric permittivity of the other rigid petrochemical-origin polyurethane materials was estimated in dependence of atmospheric parameters. These include PU foams of (a) a low density ρ_f_ ≈ 33 kg/m^3^ (lab-made), (b) a medium density ρ_f_ ≈ 274 kg/m^3^ (lab-made), (c) a medium density ρ_f_ ≈ 459 kg/m^3^ (SikaBlockM-450; Sika JSC; Baar, Switzerland), (d) a high density ρ_f_ ≈ 993 kg/m^3^ (SikaBlockM-930; Sika JSC; Baar; Switzerland), as well as monolithic polyurethanes with (a) a density ρ_p_ ≈ 1280 kg/m^3^ (lab-made) and (b) a density ρ_p_ ≈ 1351 kg/m^3^ (SikaBlockM-945; Sika JSC; Baar, Switzerland). The lab-made PU foams and monolithic polyurethane were produced according to typical rigid closed-cell PU foams formulation, given in [7]. It was experimentally shown in [7] that up to a density ≈ 550 kg/m^3^, the permittivity of Sika JSC PU foams and the lab-made PU foams is equal in the limits of uncertainties. As a material with well-known dielectric and water absorption properties, PTFE was investigated to test the methodology.

A similar methodology as the one, described in the paper for the SikaBlock^®^-M150 foams (as an example), was applied for the other materials. It was shown in [8] that a thickness of 20–25 mm ensures an appropriate penetration depth of the electrical excitation field for rigid PU foams of densities higher than 230 kg/m^3^, monolithic polyurethane, and PTFE; therefore, samples of 20 × 45 × 45 mm dimensions were used.

### 2.3. The Measured Permittivity Spectra

**Figure 1:**
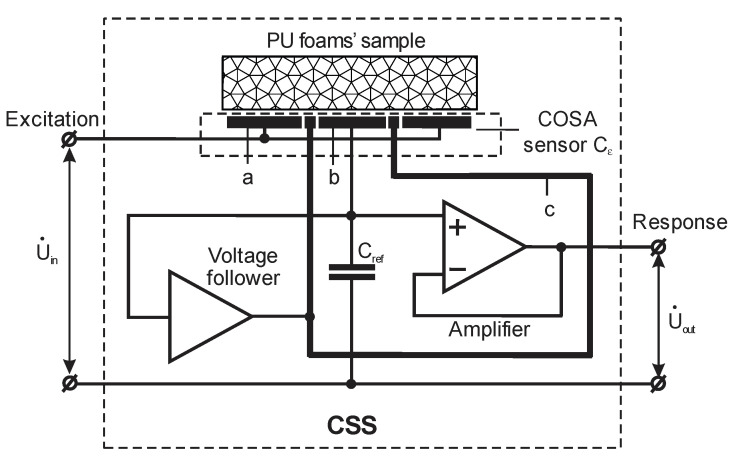
A simplified functional diagram of the capacitive one-side access sensor system; “a”—driven electrode, “b”—sensing electrode; and “c”—guard electrode.

The real part ε′(f) of the complex dielectric permittivity $\tilde{\varepsilon }$(jf) = ε′(f) − jε″(f) was measured with an innovative, experimental, dielectric spectrometer equipped with a sensor to perform non-destructive testing, at a spectrum of low frequencies of the electric field. Stray-immune capacitance measurements were made, using active guarding of the sensing electrode to increase the sensitivity and accuracy of measurements. The spectrometer consists of three main blocks: a multi-frequency excitation generator, a capacitive sensor system (CSS) of a one-side access type, and a multi-frequency response signal-processing unit [14], Figure 1.

The driven electrode “a” and sensing electrode “b” of the COSA sensor were connected to the multi-frequency excitation generator. The driven electrode was connected directly and the sensing was performed through a reference capacitor C_ref_. To carry out stray-immune capacitance measurements, the sensing electrode “b”, the reference capacitor C_ref_, and the unity gain buffer amplifier were covered with a screen, forming a guard electrode “c” on the working surface of CSS around the sensing electrode. The guard electrode was fed through a voltage follower by the same voltage as the sensing electrode. Thus, the guard electrode suppressed the electric field between the driven electrode and the sensing electrode outside the working surface of the sensor. As a result, the current in the circuit of the sensing electrode was directly proportional to the resultant electric field intensity in the test object. The voltage drop on the reference capacitor C_ref_ created by the current in the circuit of the sensing electrode formed a CSS output signal, which was passed to the multi-frequency signal-processing unit via the buffer amplifier. By suppressing the electric field between the driven electrode and the sensing electrode outside the working surface of the sensor, the solution, proposed for CSS in [11], prevented any impact of stray capacitances on the output signal and ensured that the current through the sensing electrode was proportional to the capacitance between the driven electrode and the sensing electrode. This capacitance was denoted as the sensing capacitance C_0_ when the CCS was in air; when the test object was put on the capacitive sensor without a gap, the sensing capacitance C_0_ was multiplied by the complex dielectric permittivity of the test object—the sample [14].

The foams’ sample was placed with one and the same side on the active area of the sensor, with the diameter of the annular outer electrode reaching D_0_ = 43 mm, and excited with electrodes via an electrical field generated by sinusoidal voltage signals. The amplitude value of the sinusoidal excitation signals was U_0_ = 20 V. The signals were generated at discrete frequencies, increasing in a geometric progression:

f_n_ = f_1,_ 2f_1_, ..._,_ 2^(n−1)^f_1_ Hz, where f_1_ = 10 Hz, n = 1, 2,..., 16; f = 10, 20, …, 327,680 Hz,(1)

where n—ordeal number of a frequency. The dielectric loss part ε″(f) was measured for the SikaBlock^®^-M150 PU foams with a broadband dielectric spectrometer BDS-50 (Novocontrol Technologies GmbH & Co. KG, Montabaur, Germany) in the considered frequency range 10 Hz–0.33 MHz as ε″ (f) ≈ 0.006–0.008. For monolithic polyurethane, ε″(f) = 0.042 at 1 kHz and ε″(f) = 0.088 at 0.1 MHz, and, for PTFE at frequencies f = 50 Hz–1 MHz, ε″(f) < 0.0004 [8]. The loss parts are comparatively small; therefore, $\tilde{\varepsilon }$(jf) ≈ ε′(f) and ε′(f) = ε(f) is further referred to as permittivity.

The impact of atmospheric parameters—temperature T (dry bulb), pressure (barometric) p, and relative humidity RH—on measured permittivity spectra of a sample of industrially produced, rigid, closed-cell PU SikaBlock^®^-M150 foams was investigated for one year in the test room (January–December). The permittivity of the parallelepiped-shaped test sample of dimensions 13 × 65 × 65 mm, with a density of 143.9 ≈ 144 kg/m^3^, was measured in the (1) short and (2) long measurement series (further—short series and long series) to estimate the warm-up drift of the sensor system. The spectrometer was calibrated before each measurement with regard to the measurement value, delivered by the sensor in ambient air, thus limiting the effects of moisture inside the sensor, as well as other uncertainties. A single measurement, together with calibration, took ~5 min.

At each measurement, parameters of ambient air were determined: (a) temperature and relative humidity with a thermo-hygrometer Testo 608-H1 (Testo SE & Co. KGaA, Lenzkirch, Germany), with the expanded uncertainties U95% = ±0.22 °C and U95% = ±2.2% (calibration certificate № F 5139 K19), and (b) pressure with a thermo-hygro-barometer TFA 20.3006.32 (TFA Dostmann GmbH & Co., Wertheim-Reicholzheim, Germany), with an accuracy of δp = ±5 hPa and a resolution of δδp = ±0.5 hPa. The sample, spectrometer, and appliances were situated in the test room for the entire period of investigation for thermodynamic equilibrium. No conditioning of the sample was made.

A short series was measured once every 2–3 days. After turning on the spectrometer, 3 permittivity spectra were registered consecutively in time of ~15 min. Each month, 1–2 long series were measured. After turning on the spectrometer, 30–40 permittivity spectra were registered consecutively in time of ~200 min. Altogether, 145 short and 16 long series were measured.

The permittivity spectra of each short series were compared and those with considerable deviations from typical were omitted. For each short series, an averaged spectrum was calculated at each frequency:

(2) $$\varepsilon_{\mathrm{av}}(f_{n})=1/3\;\sum_{i=1}^{3}\varepsilon_{i}\left(f_{n}\right),n=1,2,...,16;$$

where n = (logf_n_ − 1)/log2 + 1, as shown in Equation (1). The averaged spectrum was approximated by a 2^nd^-order polynomial to outline the main trends:

ε_av_(f_n_) = A_1n_n^2^ + B_1n_n + C_1n_; (3)

where A_1n_, B_1n_, and C_1n_ are the numerical coefficients. Altogether an amount of K = 134 of typical, averaged and approximated spectra was amassed. The temperature, relative humidity, and pressure varied insignificantly during a single short series and average values of T, p, or RH were calculated for each short series:

(4) $$y_{\mathrm{av}}=1/3\;\sum_{i=1}^{3}y_{i},$$

where y—values of T, p, or RH during the measurement of a single spectrum. ε_av_(f_n_, k), T_av_, p_av_, and RH_av_ are further referred to as ε(f_n_, k), T, p, and RH for simplicity. Variations in the permittivity, temperature, relative humidity, and pressure in the one-year-long investigation were approximated by 3rd-order polynomials. Trends in variations of ε, T, p, and RH were compared and conclusions were made. To estimate the correlation between the random variables (permittivity and atmospheric parameters), charts of variables’ pairs, i.e., “ε(f_n_)—T”, “ε(f_n_)—p”, and “ε(f_n_)—RH”, were plotted and the correlations between ε(f_n_) and T, p, and RH were modelled by polynomials:

ε(f_n_, y) = A_2n_y^2^ + B_2n_y + C_2n_, (5)

where y—T, p, or RH, and A_2n_, B_2n_, and C_2n_—numerical coefficients. The corresponding coefficients of determination R^2^ were calculated and compared.

The spectra of each long series were compared and those with considerable deviations from a typical spectrum were omitted. The typical long series were approximated by polynomials:

ε(f_n_, m) = A_3n_m^2^ + B_3n_m + C_3n_, m = 1, 2,..., M; (6)

where A_3n_, B_3n_, and C_3n_—numerical coefficients, m—the ordeal number of a permittivity value in a long series, and M—the total number of spectra in a long series. Altogether, J = 16 series of typical approximated spectra were amassed. The first three permittivity values in a long series were considered as a separate short series, and the corresponding average ε_av3_(f_n_) was calculated:

(7) $$\varepsilon_{\mathrm{av}3}(f_{n})=1/3\;\sum_{m=1}^{3}\varepsilon_{m}(f_{n}),$$

The temperature, relative humidity, and pressure varied insignificantly during a long series and the average values of T, p, or RH were calculated for each long series:

(8) $$y_{\mathrm{av}}=1/M\;\sum_{m=1}^{M}y_{m},$$

where y—values of T, p, or RH during the measurement of a single spectrum. T_av_, p_av_, and RH_av_ are further referred to T, p, and RH for simplicity.

### 2.4. Water Vapor in Air

Relative humidity (RH) indicates the relative amount of moisture in air as a percentage of the maximum amount m_wmax_ (at saturation, below 100 °C, pressure 1 atm), that can be mixed with the air at a specific temperature and pressure:

RH = m_w_/m_wmax_. (9)

The value of RH provides a consistent measurement of humidity only if it is combined with the corresponding temperature. It is of interest to link the measured values of permittivity to a physical quantity, characterising the absolute moisture content in a unit volume of air in the test room at a certain temperature and relative humidity; therefore, the absolute amount of water vapor m_w_ in air (g/m^3^) at saturation (RH = 100%) was considered. The empirical tabulated values of the maximum absolute amount of water vapor m_wmax_ in air (g/m^3^) at saturation (RH = 100%) [23] in the practically useful temperature range 0 °C ≤ T ≤ 30 °C are exponentially linked to temperature:

m_wmax_ = ae^bT^; where a = 5.4086 and b = 0.0547. (10)

Then, the water vapor mass in air at temperatures 0 °C ≤ T ≤ 30 °C can be expressed:

m_w_ = RH m_wmax_ = RH ae^bT^. (11)

To estimate the correlation between the measured values of permittivity ε(f_n_) in the short series and water vapor mass in a cubic meter of air m_w_, plots of random variables’ pairs “ε(f_n_)—m_w_” were constructed for the 16 frequencies. The correlation between ε(f_n_) and the calculated m_w_ values at each frequency was modelled by 2^nd^-order polynomials:

ε(f_n_, m_w_) = A_4n_m_w_^2^ + B_4n_m_w_ + C_4n_, (12)

where A_4n_, B_4n_, and C_4n_—numerical coefficients.

Numerical calculations showed that water vapor mass in a cubic meter of air m_w_ changed insignificantly (Section 3.3) during the measurement each of the long series; therefore, it was assumed for a long series:

(13) $$m_{w}=m_{\mathrm{wav}}=1/M\;\sum_{m=1}^{M}m_{\mathrm{wm}}\approx \mathrm{const}.,$$

where m_wm_—the value of m_w_ at the m-th spectrum. Then, the plateau of permittivity values in the j-th long series can be defined as a set of MP_j_ ≥ 10 consecutive data points with a straight trendline of an approximating equation ε^p^_j_(f_n_) ≈ const. The plateau data points are considered to be acquired in a stabile measurement process and the permittivity’s plateau value can be calculated for each long series:

(14) $$\varepsilon^{p}(f_{n})=1/\mathrm{MP}\;\sum_{\mathrm{mp}=1}^{\mathrm{MP}}\varepsilon_{\mathrm{mp}}\left(f_{n}\right),$$

where mp = 1, 2, …; MP is the ordeal number of a data point in the plateau. Consequently, the warm-up drift can be calculated:

Δε(f_n_) = ε_av3_(f_n_) − ε^p^(f_n_). (15)

as the difference between the averaged first three permittivity values of a long series and the plateau value.

### 2.5. The True Permittivity

As a physical property, the true dielectric permittivity of closed-cell PU foams has to be independent of air humidity. The measured permittivity values, most close to the true permittivity, can be expected in measurements in dry air, when m_w_ → 0.0 g/m^3^. In both the short and long series, the registered water vapor mass in a cubic meter of air varied in the limits 3.1 g/m^3^ ≤ m_w_ ≤ 11.1 g/m^3^. To estimate the true dielectric permittivity of SikaBlock^®^-M150 PU foams, the permittivity spectra were calculated, as shown in Equation (12), at discrete values m_w_ = 0.0, 1.0, 2.0, …, 11.0 g/m^3^:

m_wi_ = (i − 1)Δm_w_, (16)

where Δm_w_ = 1.0 g/m^3^ and i = 1, 2, …, 12. The spectra were calculated as well at (1) standard reference conditions [ISO 5011] T = 20 °C, p = 1013.25 hPa, and RH = 50%, when m_w_ = 8.05 g/m^3^ and (2) the average m_wav_ = 6.70 g/m^3^ over the entire 134 short series. Since the warm-up drift Δε(f_n_) exhibited no dependence of m_w_ and remained nearly constant for all m_w_ = 4 … 11 g/m^3^, the true permittivity spectrum was determined as the plateau values of calculated permittivity spectrum at dry air:

ε^T^(f_n_, 0.0) = ε(f_n_, 0.0) − Δε(f_n_). (17)

Comparative measurements were made with the dielectric spectrometer BDS-50, comprising a parallel plate capacitor, for a cylindrical SikaBlock^®^-M150 sample, diameter D = 29.9 mm, height h = 2.0 mm, and density ρ = 146 kg/m^3^. Three consecutive measurements of the permittivity spectra ε(f_n_) were made at each of the 10 short series; simultaneously, parameters T, p, and RH were determined in the test room. The spectra were estimated, sorted, and averaged over each series and approximated by 2nd-order polynomials. The sample and spectrometer were situated in the test room to investigate the thermodynamic equilibrium. No conditioning of the sample was made.

### 2.6. Measurement Uncertainties

The uncertainty of the measured permittivity was evaluated following the ISO “Guide to the Expression of Uncertainty in Measurement” (GUM) [27,28]. The experimentally identified range of water vapor values 3.0 g/m^3^ ≤ m_w_ ≤ 12.0 g/m^3^ was divided into 9 intervals of width Δm_w_ = 1.0 g/m^3^, as demonstrated in Table 1:

Δm_w_*(i + 2) ≤ m_w_ < Δm_w_*(i + 3), i = 1, 2, …, 9. (18)

The K = 134 spectra of the short series were sorted according to the corresponding m_w_ values:

(19) $$K=\sum_{i=1}^{9}K_{i}=134;$$

where K_i_—the number of spectra, corresponding to m_w_ values of the i-th interval.

The permittivity spectra were considered as measured at the standard reference conditions, when the corresponding atmospheric parameters fell into the limits T = 20 ± 2 °C, RH = 50%, and p = 1013 hPa ± 3 hPa. The average value ε_avi_, the standard deviation σ, and the expanded uncertainty U95% were estimated for the K_i_ permittivity spectra of short series, corresponding to the i-th water vapor mass interval:

(20) $$\varepsilon_{\mathrm{avi}}(f_{n})=1/K_{i}\;\sum_{j=1}^{\mathrm{Ki}}\varepsilon_{j}\left(f_{n}\right),n=1,2...,16;$$

(21) $$\sigma_{i}(f_{n})=\sqrt{\sum_{j=1}^{K_{i}}{\left[\varepsilon_{\mathrm{ij}}\left(f_{n}\right)-{\;\varepsilon }_{\mathrm{avi}}\left(f_{n}\right)\right]}^{2}/\left(K_{i}-1\right)}$$

U95%_i_(f_n_) = ±k_i_ σ_i_(f_n_), (22)

where i = 1, 2,..., 9; j = 1, 2, …, K_i_ and k_i_ is the coverage coefficient for the i-th interval. When K_i_ ≥ 10, the reliability criterion is considered to be met, and the coverage coefficient is assumed as k_i_ = 2.0 and the expanded uncertainty is estimated as U95%_i_(f_n_) = ±2.0σ_i_(f_n_) [27].

To estimate uncertainties in the plateau region, 9 series with the longest plateaus were selected from the J = 18 long series in such a way that ensured one series in each m_w_ interval 3.0 ≤ m_w_ < 4.0, 4.0 ≤ m_w_ < 5.0, …, 11.0 ≤ m_w_ < 12.0 g/m^3^. The uncertainty of MP_i_ permittivity values corresponding to the plateau of the i-th long series was estimated in a similar way as for the short series.

## 3. Results and Discussion

### 3.1. Permittivity in the Short Series

**Figure 2:**
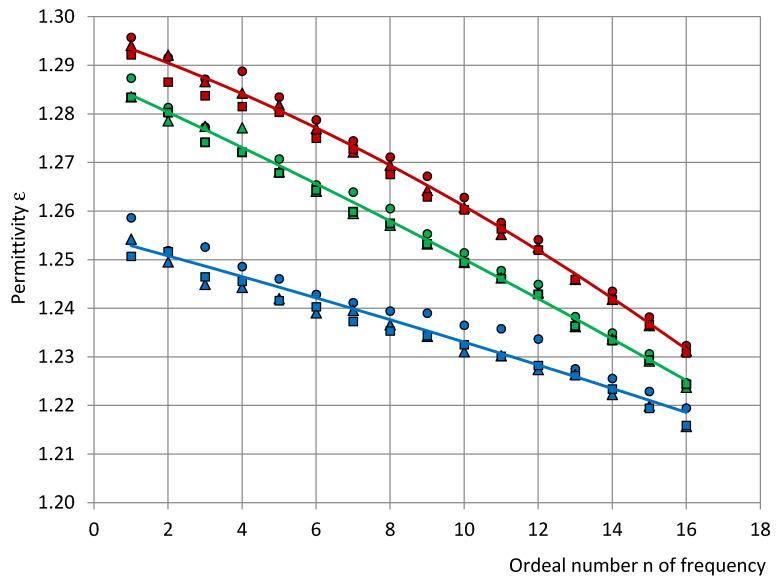
The permittivity spectra of PU SikaBlock^®^-M150 foams (density of 144 kg/m^3^) at a relative humidity of atmosphere in the test room RH: (1) 21% (blue), (2) 50% (green), and (3) 60% (red). Markers—data points of the three permittivity spectra of a short series; the continuous lines—the averaged and approximated permittivity spectra.

Figure 2 gives examples of short series at (1) the lowest identified relative humidity RH = 21% (middle of April, T = 22.4 °C, and p = 1029 hPa), (2) the standard reference conditions RH = 50% (middle of October, T = 20 °C, and p = 1019 hPa), and (3) the highest identified RH = 60% (end of July, T = 18.2 °C, and p = 1016 hPa).

**Figure 3:**
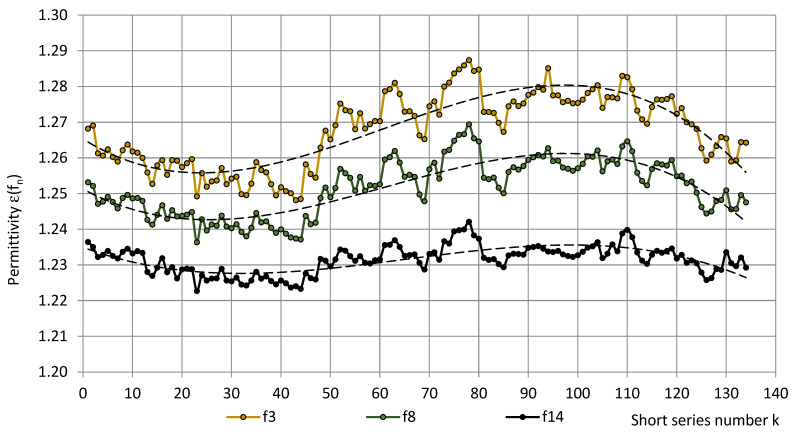
The permittivity ε of PU SikaBlock^®^-M150 foams (density of 144 kg/m^3^) in the short series at frequencies f_3_ = 40 Hz (beige), f_8_ = 1280 Hz (green), and f_14_ = 81,920 Hz (black).

The main trends of permittivity in the short series, depicted in Figure 3, e.g., at frequencies f_3_ = 40 Hz, f_8_ = 1280 Hz, and f_14_ = 81,920 Hz (roughly f_3_ ≈ 100 Hz, f_8_ ≈ 1000 Hz, and f_14_ ≈ 100,000 Hz), were described by the approximating equations:

ε(f_3_) = −0.00000012k^3^ + 0.00002217k^2^ − 0.00085441k + 1.26576007, R² = 0.74, ε(f_8_) = −0.00000010k^3^ + 0.00001810k^2^ − 0.00073269k + 1.25149051, R² = 0.71 and ε(f_14_) = −0.00000006k^3^ + 0.00001078k^2^ − 0.00051596k + 1.23458232, R² = 0.52; (23)

where k = 1, 2,..., 134 and R^2^—the coefficient of determination. At other frequencies, the graphs were similar.

**Figure 4:**
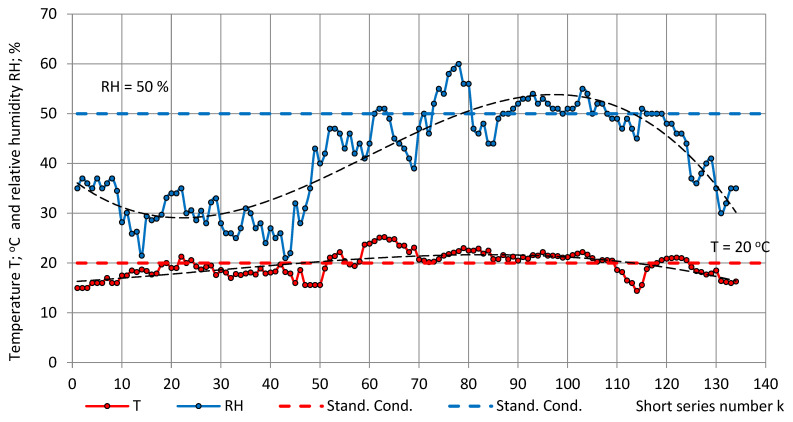
Atmospheric temperature T (red) and relative humidity RH (blue) in the test room in the short series.

**Figure 5:**
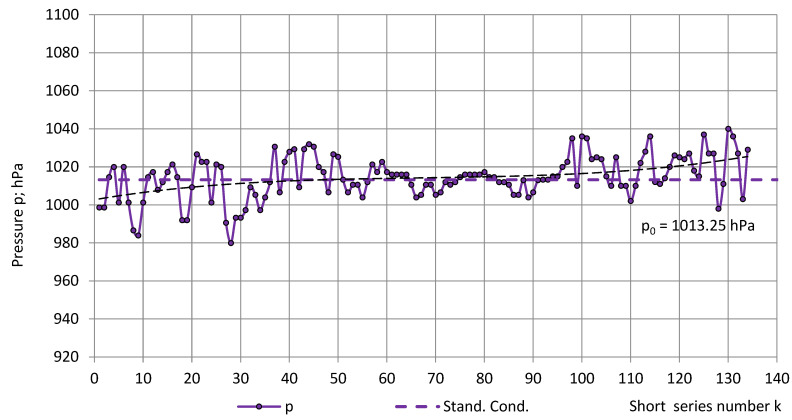
Atmospheric pressure p in the test room in the short series.

The temperature, relative humidity, and pressure in the short series are depicted in Figure 4 and Figure 5. The main trends of experimental data were approximated by third-order polynomials:

T = −0.000009k^3^ + 0.000804k^2^ + 0.063931k + 16.242152, R^2^ = 0.48, RH = −0.000117k^3^ + 0.020929k^2^ − 0.754723k + 36.793538, R² = 0.80 and p = 0.000029k^3^ − 0.005929k^2^ + 0.436991k + 1002.709884; R² = 0.17. (24)

In the short series, the relative humidity varied in the limits of low to medium values 21% ≤ RH ≤ 60%. The average temperature and relative humidity were calculated as T_av_ = 19.6 °C ± 2.5 °C (13%) and RH_av_ = 41% ± 10% (24%), respectively.

The average pressure in the short series was calculated as p_av_ = 1014 hPa ± 11 hPa (1%). The value was close to the pressure at standard reference conditions p_0_ = 1013.25 hPa (1 atm) and a coefficient of variation v ≈ 1%; therefore, it was further assumed as p ≈ const. ≈ p_0_.

**Figure 6:**
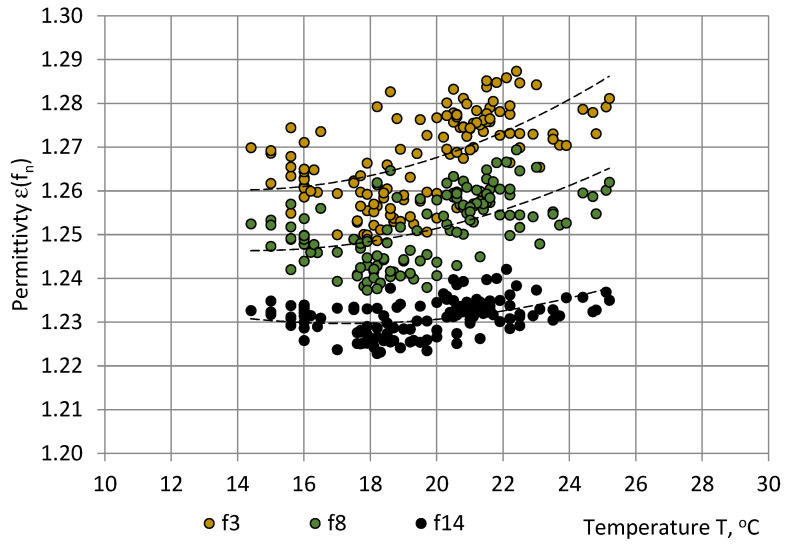
Correlation between the permittivity ε of PU SikaBlock^®^-M150 foams (density of 144 kg/m^3^) and the atmospheric temperature T in the test room at frequencies f_3_ = 40 Hz (beige), f_8_ = 1280 Hz (green), and f_14_ = 81,920 Hz (black).

**Figure 7:**
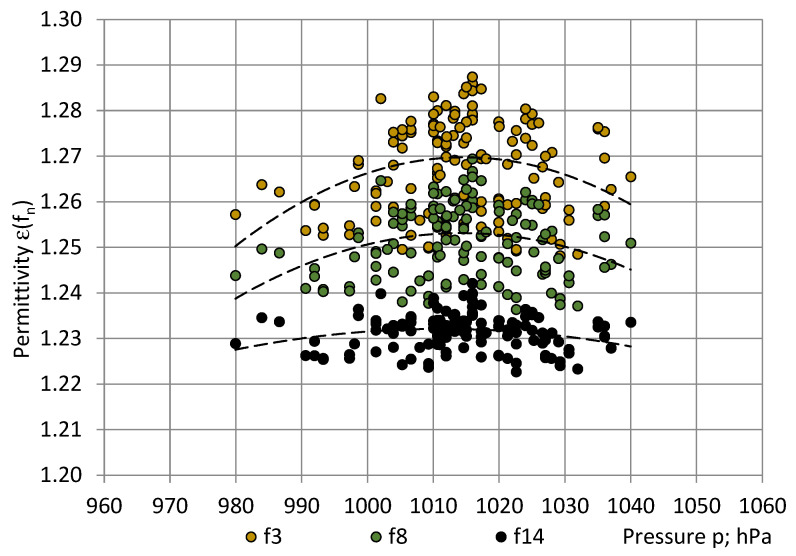
Correlation between the permittivity ε of PU SikaBlock^®^-M150 foams (density of 144 kg/m^3^) and the atmospheric pressure p in the test room at frequencies f_3_ = 40 Hz (beige), f_8_ = 1280 Hz (green), and f_14_ = 81,920 Hz (black).

**Figure 8:**
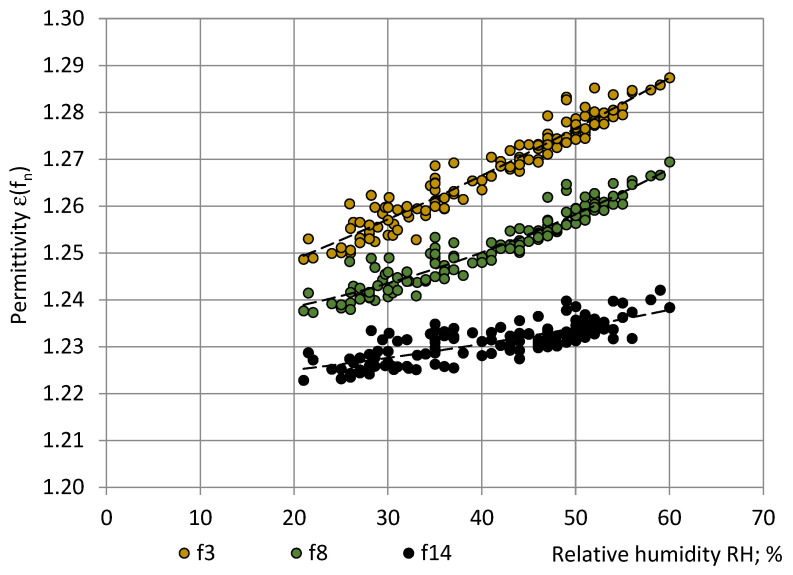
Correlation between the permittivity ε of PU SikaBlock^®^-M150 foams (density of 144 kg/m^3^) and the relative humidity RH of atmosphere in the test room at frequencies f_3_ = 40 Hz (beige), f_8_ = 1280 Hz (green), and f_14_ = 81,920 Hz (black).

Figure 3, Figure 4 and Figure 5 show that the main trend of permittivity the most closely follows that of relative humidity. Plots of the random variable pairs “ε—T”, “ε—p”, and “ε—RH” are given in Figure 6, Figure 7 and Figure 8. The correlation between variables is modelled by Equations (25)–(27).

ε(f_3_) = 0.000209T^2^ − 0.005870T + 1.301465; R² = 0.35; ε(f_8_) = 0.000161T^2^ − 0.004645T + 1.279791; R² = 0.31 and ε(f_14_) = 0.000129T^2^ − 0.004474T + 1.268358; R² = 0.19. (25)

ε(f_3_) = −0.000016p^2^ + 0.031990p − 14.962271; R² = 0.09; ε(f_8_) = −0.000012p^2^ + 0.024356p − 11.100558; R² = 0.09 and ε(f_14_)= −0.000006p^2^ + 0.012337p − 5.009426; R² = 0.08. (26)

ε(f_3_) = 0.000004RH^2^ + 0.000675RH + 1.233580; R² = 0.93; ε(f_8_) = 0.000008RH^2^ + 0.000110RH + 1.233174; R² = 0.91; ε(f_14_) = 0.000002RH^2^ + 0.000147RH + 1.221239; R² = 0.63. (27)

By comparing the R^2^ values, the best correlation was identified between the PU foams permittivity and the relative humidity (0.63 ≤ R^2^ ≤ 0.93), as shown in Table 2. The correlation with temperature was small (0.19 ≤ R^2^ ≤ 0.35) and practically no correlation with pressure was revealed (0.08 ≤ R^2^ ≤ 0.09).

### 3.2. Water Vapor in Air

**Figure 9:**
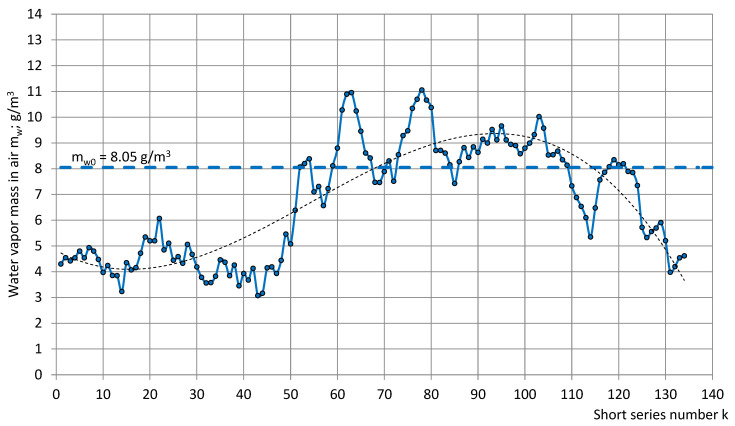
Water vapor mass m_w_ in the air of the test room in the short series.

The calculated water vapor mass in the air m_w_ in 134 short series is given in Figure 9.

Figure 9 shows that, in the short series, water vapor mass in a cubic meter of air varied in the limits 3.1 g/m^3^ ≤ m_w_ ≤ 11.1 g/m^3^, where m_w0_ = 8.05 g/m^3^ corresponds to standard reference conditions. In a year-long observation, the upper detected value of m_w_ in the experiment premises was nearly four times higher than the lower value.

**Figure 10:**
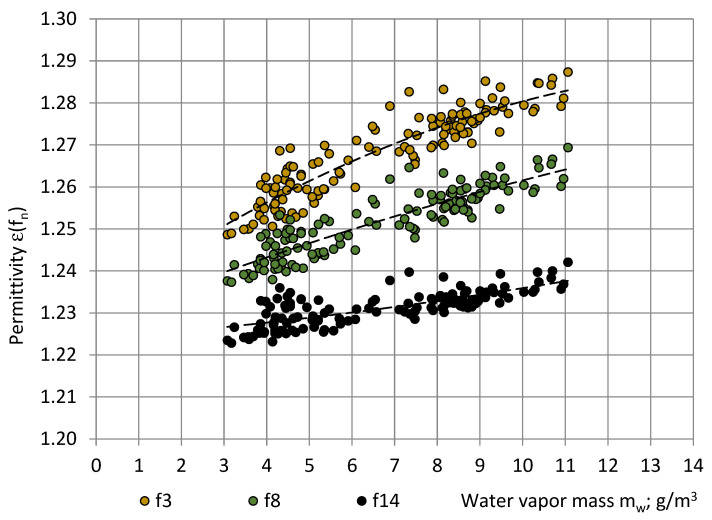
Correlation between the permittivity ε_av_ of PU SikaBlock^®^-M150 foams (density of 144 kg/m^3^) and the water vapor mass m_w_ in the air of the test room at frequencies f_3_ = 40 Hz (beige), f_8_ = 1280 Hz (green), and f_14_ = 81,920 Hz (black).

Figure 10 gives plots of variables “ε_av_(f_n_)—m_w_”. The corresponding correlations are described by the following equations:

ε(f_3_) = −0.00022m_w_^2^ + 0.00708m_w_ + 1.23146; R² = 0.85; ε(f_8_) = −0.00007m_w_^2^ + 0.00400m_w_ + 1.22828; R² = 0.80; ε(f_14_) = 0.00004m_w_^2^ + 0.00079m_w_ + 1.22385; R² = 0.57. (28)

In a practically useful temperature range of 0 °C ≤ T ≤ 30 °C, Equation (28) can be used to calculate the permittivity spectra of PU SikaBlock^®^-M150 foams at any m_w_ value in the limits 3.1 g/m^3^ ≤ m_w_ ≤ 11.1 g/m^3^, as well as to extrapolate permittivity values beyond the given limits. The correlation between ε(f_n_) and m_w_ was quite high: 57% ≤ R² ≤ 85%. It can be concluded that the permittivity at lower frequencies (10–100 Hz) is more sensitive to air humidity; as a result, it increases more rapidly with an increase in m_w_ than permittivity at higher frequencies. The increase may be attributed to an increase in the number of ionic charge carriers, generated by water molecules in polyurethane [26].

### 3.3. Permittivity in the Long Series

**Figure 11:**
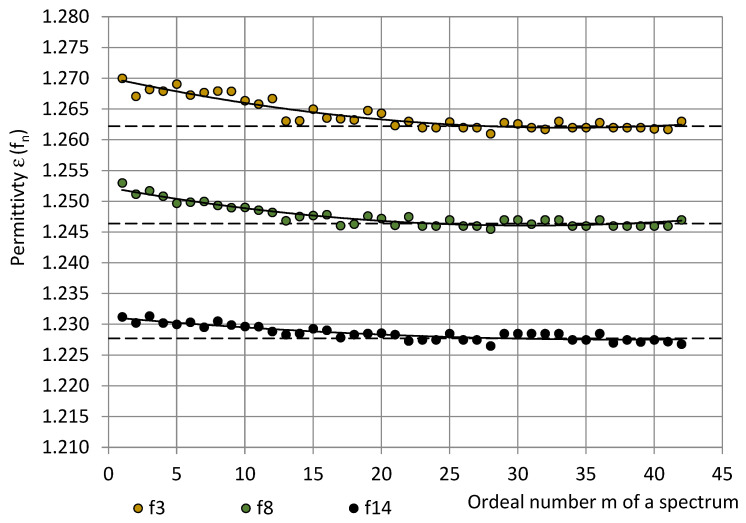
The permittivity of PU SikaBlock^®^-M150 foams (density of 144 kg/m^3^) in a long series at frequencies f_3_ = 40 Hz (beige), f_8_ = 1280 Hz (green), and f_14_ = 81,920 Hz (Black).

Figure 11 gives an example of permittivity in a long series (t = 3 h, 30 min, and m = 42 spectra). During the series, the temperature changed from 16.2 °C to 16.4 °C and the relative humidity changed from 44% to 45%, and the pressure remained constant ≈ 995 hPa. As a result, the water vapor mass in air increased insignificantly from 5.8 g/m^3^ to 6.0 g/m^3^. The same trend was observed for all typical long series; in the majority of series, the RH remained constant. It can be seen that starting from around m = 21, when the spectrometer was turned on for 1 hour and 45 min (The warm-up time), the measured permittivity values stabilised and further fluctuated around the plateau value (21 ≤ m ≤ 42): ε^p^(f_3_) ≈ 1.262, ε^p^(f_8_) ≈ 1.246, and ε^p^(f_14_) ≈ 1.228. The warm-up drift was estimated as the difference between the averaged first three permittivity values and the plateau value, as shown in Equation (15):

Δε(f_3_) ≈ 0.006, Δε(f_8_) ≈ 0.005 and Δε(f_14_) ≈ 0.003. (29)

**Figure 12:**
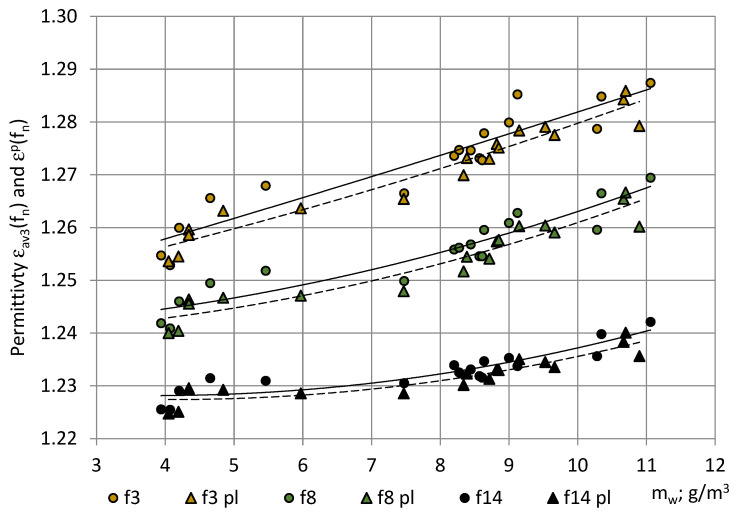
The dependence of permittivity of PU SikaBlock^®^-M150 foams (density of 144 kg/m^3^) in the long series on water vapor mass m_w_ in the air of the test room at frequencies f_3_ = 40 Hz (beige), f_8_ = 1280 Hz (green), and f_14_ = 81 920 Hz (black). (1) Bullets—average permittivity of the first three measurements ε_av3_(f_n_) and (2) triangles—the plateau values ε^p^(f_n_).

After turning on the spectrometer, the sensor system’s parts warmed up and expanded thermally until reaching an operational temperature, and the water condensate evaporated from surfaces. When the processes achieved equilibrium, a plateau appeared in the long-series permittivity values. The averaged permittivity values of the first three measurements of the long series and the plateau values are given in Figure 12 for the typical long series, together with the corresponding trendlines. During the long series, the water vapor in air increased for less than m_w_ ≈ 0.5 g/m^3^.

The permittivity values in a long series were approximated by second-order polynomials:

ε(f_3_) = 0.000007m^2^ − 0.000483m + 1.270099; R² = 0.88; ε(f_8_) = 0.000007m^2^ − 0.000401m + 1.252235; R² = 0.89 and ε(f_14_) = 0.000002m^2^ − 0.000192m + 1.231158; R² = 0.78. (30)

It can be seen that the trendlines of averaged values of the first three measurements in the long series are similar to the trendlines of the dedicated short series, as shown in Figure 10. The trendlines of plateau values ε^p^(f_n_) are similar as well, only at smaller values: ε^p^(f_n_) < ε(f_n_). The warm-up drift Δε(f_n_) exhibited no dependence of m_w_ and remained nearly constant for all m_w_ = 4 … 11 g/m^3^: Δε(f_3_) ≈ 0.0025–0.0033, Δε(f_8_) ≈ 0.0020...0.0025, and Δε(f_14_) ≈ 0.0015...0.0020. The identified warm-up drift might be caused by the thermal expansion at warm-up of the sensor system’s parts. The warm-up time of the spectrometer to reach stable readings remained nearly constant at all detected m_w_ values: 1 h, 45 min–1 h, and 50 min.

### 3.4. The True Permittivity

**Figure 13:**
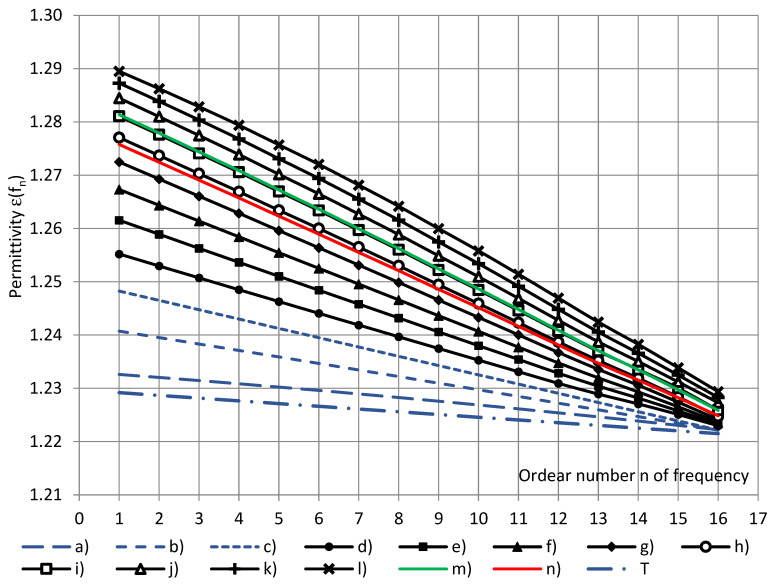
The calculated permittivity spectra of PU SikaBlock^®^-M150 foams (density of 144 kg/m^3^) at water vapor mass in air of the test room m_w_: (a–c) m_w_ = 0.0, 1.0, and 2.0 g/m^3^ (blue, extrapolated); (d–l) m_w_ = 3.0, 4.0, …, 11.0 g/m^3^; green—at the standard reference conditions m_w_ = 8.05 g/m^3^, red—at the average m_wav_ = 6.70 g/m^3^ and “T”—the true permittivity spectrum (“n”—the ordeal number of frequency).

The permittivity spectra, calculated at discrete water vapor mass values m_w_ = 0.0, 1.0, 2.0, …, 11.0 g/m^3^, are given in Figure 13 together with spectra, calculated at (1) the standard reference conditions (m_w_ = 8.05 g/m^3^) and at (2) the average m_wav_ = 6.70 g/m^3^ over the entire short series. The permittivity spectrum, calculated at m_w_ = 0.0 g/m^3^ (dry air), is considered to be the most close to the true permittivity spectrum of closed-cell PU foams. Taking into account Equation (28) and the numerical values of Δε(f_n_), the true permittivity spectrum was calculated as the plateau values of the averaged permittivity spectrum at dry air, as shown in Figure 13 (spectrum “T”). 

In the comparative measurements with the spectrometer BDS-50, the water vapor mass in the air covered a range 4.0 g/m^3^ < m_w_ < 9.0 g/m^3^. The permittivity, measured in the short series with BDS-50 and the COSA sensor-based measurement system, differed for less than ±0.7% at similar m_w_ values, demonstrating a high accuracy of the latter. The permittivity, measured with spectrometer BDS-50, exhibited similar dependence on water vapor mass in air as the permittivity, measured in the short series with a COSA sensor, as shown in Figure 10.

### 3.5. Measurement Uncertainties

The uncertainties of permittivity spectra of short series, corresponding to the nine intervals of m_w_ values, are summarized in Table 3 for frequencies with odd ordeal numbers n = 1, 3, …, 15 (at even values of “n”, the trends remain similar). In all intervals, except the seventh, the number of spectra K_i_ > 10, the coverage coefficient k_i_ = 2.0, and the expanded uncertainty can be calculated as U95%_i_(f_n_) = ±2σ_i_(f_n_). In the seventh interval U95%_i_(f_n_) ≥ ±2σ_i_(f_n_), the main sources of uncertainty comprised the spectrometer itself [8], the chosen final width of the humidity interval Δm_w_ = 1 g/m^3^, the uncertainty of T and RH values, the uncertainty of air gaps between the sample and the sensor [8], etc.

In each of the nine long series, the number of data points in plateau was MP_i_ > 10. Then, for permittivity values at plateau, U95%_i_(f_n_) = ±2σ_i_(f_n_). The value of U95%^p^, at a certain frequency, was ≈ 4–8 times smaller than that in the short series. More experimental long series at different water vapor masses in a cubic meter of air m_w_ would be necessary to narrow the estimated limits of uncertainty of permittivity data from the plateau.

The statistical characteristics of atmospheric parameters T, RH, and p in the nine intervals of water vapour mass in air m_w_, registered in the short series, are given in Table 4.

### 3.6. Other Materials

**Figure 14:**
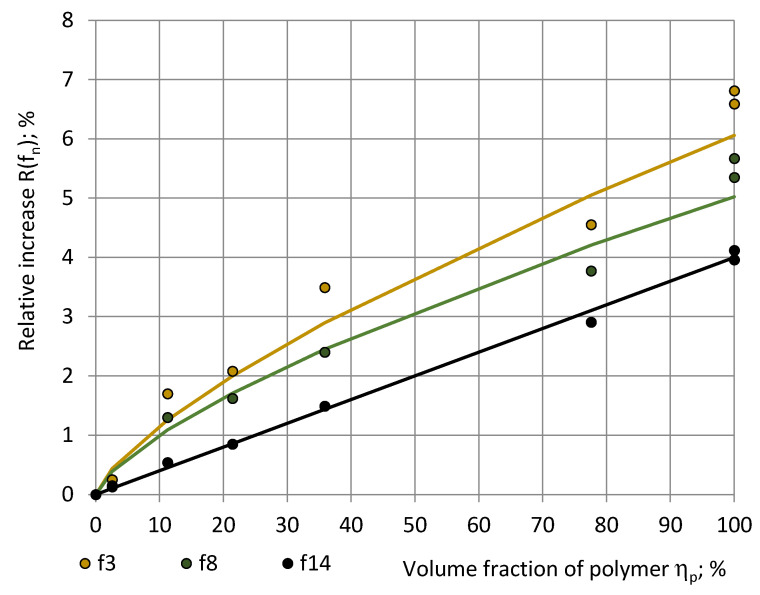
The relative increase R(f_n_) in the true permittivity of rigid polyurethane materials in dependence of volume fraction of polyurethane at frequencies f_3_ = 40 Hz (beige), f_8_ = 1280 Hz (green), and f_14_ = 81,920 Hz (black).

The other rigid polyurethane materials—foams and monolithic polyurethanes—exhibited a similar dependence of the measured permittivity value on water vapour mass in the air of the test room as PU SikaBlock^®^-M150 foams. A relative increase R(f_n_) in the measured permittivity value was estimated for PU materials as:

R(f_n_) = [ε(f_n_; 8 g/m^3^) − ε(f_n_; 3 g/m^3^)]/ε(f_n_; 3 g/m^3^), (31)

at an increase of m_w_ value from 3 g/m^3^ in very dry premises to a value at standard reference conditions m_w_ ≈ 8 g/m^3^, Figure 14. Considering the permittivity at 3 g/m^3^ as sufficiently close to the true permittivity value, R(f_n_) characterised a relative increase in the true permittivity value upon an increase in m_w_ to the value, corresponding to standard reference conditions.

It can be seen that R(f_n_) increases with an increase in the volume fraction η_p_ = ρ_f_/ρ_p_ of monolithic plastic following a power law R(f_n_) = C*(η_p_)^n^, where C = 0.22, 0.20, and 0.18 and n = 0.72, 0.70, and 0.62 at f_3_, f_8_, and f_14_. The relative increase R(f_n_) in the true permittivity remained less than 7% at f_3_, f_8_, and f_14_. The character of the relationship R(f_n_) = R(f_n_; η_p_) might be explained with a combined effect of structure of the PU foams at different η_p_ and the characteristic water absorption depth in polyurethane at different RH and T values. More mathematical modelling would be necessary in that direction. The density of monolithic polyurethanes differs slightly, depending on the chemical formulation; therefore, a common value ρ_p_ = 1 280 kg/m^3^ was used to calculate the η_p_ of all PU foams, as shown in Table 5.

The experimental data of PTFE showed no impact of water vapour mass in ambient air on the measured permittivity value. For all 3.0 ≤ m_w_ ≤ 8.0 g/m^3^ and at all considered frequencies f = 10, 20, …, 327,680 Hz permittivity of PTFE remained practically constant ε ≈ 2.10 ± 0.011 (U95%); consequently, R(f_n_) = 0%. In other words, no water absorption and no dielectric dispersion were detected, which corresponds with well-known properties of PTFE. The experimental setup and methodology provided proper results for the PTFE test material.

## 4. Theoretical

### 4.1. Permittivity of Air

The permittivity of air in the test room as a function of atmospheric parameters in the same was estimated by an empiric relation:

(32) $$\varepsilon_{\mathrm{air}}=\varepsilon_{0}\;\left[1+\frac{211}{T}\;\left(p+\frac{48\;p_{s}}{T}\;RH\right)10^{-6}\right];$$

where ε_0_—the permittivity of vacuum, T—the temperature (K), RH—the relative humidity (%), p—the pressure (mm Hg), and p_s_— the pressure of saturated water vapor at temperature T (a tabulated experimental relationship (mm Hg)) [17]. The dependence of air permittivity on frequency is not taken into account. In the temperature range of interest 273.15 K ≤ T ≤ 303.15 K (0 °C ≤ T ≤ 30 °C), the experimental dependence of p_s_ on temperature can be modelled with an exponential function:

p_s_ = 3.325⋅10^−9^e^0.0605045T^, (33)

where p_s_ is calculated in standard atmospheres (atm) and T is calculated in Kelvin degrees.

**Figure 15:**
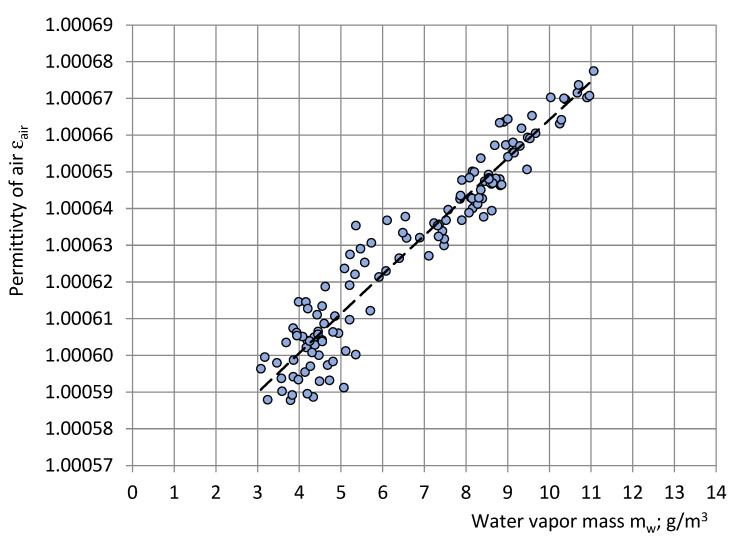
Correlation between the permittivity ε_air_ of air in the test room and water vapour mass m_w_ of air in the test room (short series).

Expressing p_s_ in mm Hg (1 atm = 760 mmHg at 273.15 K), the permittivity of air in the test room as a function of water vapor mass in the same m_w_ (g/m^3^) was estimated for the short series, as shown in Figure 15:

ε_air_ = 0.000011m_w_ + 1.000558. (34)

According to the trendline, water vapor mass in air at the entire measurement series varied in the limits 3.0 ≤ m_w_ ≤ 11.0 g/m^3^ and permittivity of air 1.000591 ≤ ε_air_ ≤ 1.000675. When m_w_ = 0.0 g/m^3^ (dry air), then ε_air_ ≈ 1.000558. The driest air in the test room was identified in March, April, and May: m_waver_ ≈ 3.6 g/m^3^. The highest wetness was identified in June, July, and August: m_waver_ ≈ 10.5–11.1 g/m^3^. This corresponds to long-term open-air meteorological observations in Riga, Latvia, where the driest month is April and the wettest months are August and July.

### 4.2. Permittivity of an Open-Cell PU Foams Sample

The SikaBlock^®^-M150 foams sample comprises three horizontal layers: (1) the top and bottom layers of cut cells where the air of the test room substituted the technological foaming gas and (2) the middle layer of ~98% closed cells filled with the mentioned gas. The thickness of the top and bottom layers approximately equals the average dimension of cells D_aver_ ≈ 0.20–0.25 mm at a PU foam density of 150 kg/m^3^, depending on chemical formulation [1,29,30]. The thickness of the middle layer was calculated by h_m_ ≈ h − 2D_aver_ = 20 mm − 2 × 0.25 mm ≈ 19.50 mm. The top layer lies beyond the penetration depth of the electric field and has no impact on the permittivity measurements. The permittivity of the open-cell bottom layer, situated in the direct vicinity of the annular electrodes of the COSA sensor, where the fast attenuating electric field is the most intensive, was estimated in dependence of the water vapor mass in the ambient air.

It was assumed that the entire sample undergoes reticulation, e.g., by leaching the thin cellular walls. By neglecting the volume fraction of the polymer in the cell walls as comparatively small, we obtain an open-cell PU foams sample filled with the ambient air, of the same size and volume fractions of components as the closed-cell one. The permittivity of the open-cell PU foam sample, comprising humid air, must be evaluated. The Maxwell–Garnett equation

(35) $$\varepsilon_{f}\left(f_{n}\right)={\;\varepsilon }_{p}\left(f_{n}\right)+\frac{{3\eta }_{a}\varepsilon_{p}\left(f_{n}\right)\left[\varepsilon_{a}-{\;\varepsilon }_{p}\left(f_{n}\right)\right]}{\left[\varepsilon_{a}+2\varepsilon_{p}\left(f_{n}\right)-\eta_{a}\left(\varepsilon_{a}-{\;\varepsilon }_{p}\left(f_{n}\right)\right)\right]},$$

was applied in [7] to calculate the effective macroscopic permittivity of rigid PU foams as a “Polymer-air” composite, where ε_p_(f_n_) and ε_a_ represent the true permittivity of monolithic polyurethane and air. The volume fractions of the monolithic polymer and air in PU foams equal:

(36) $$\eta_{p}={\;V}_{p}/V_{f}=(m_{p}/\rho_{p})/\left(m_{f}/\rho_{f}\right)={\;\rho }_{f}/\rho_{p}\;and\;\eta_{a}={\;V}_{a}/V_{f}=\left(V_{f}-{\;V}_{p}\right)/V_{f}=1-{\;V}_{p}/V_{f}=1-{\;\rho }_{f}/\rho_{p};$$

where V_f_, V_p_, and V_a_ represent the volume of foams, the monolithic polymer, and the air. The density of the monolithic polyurethane SikaBlock^®^-M960 was measured as ρ_p_ = 1180 kg/m^3^. Then, for PU SikaBlock^®^-M150 foams with a density of 144 kg/m^3^, volume fractions of monolithic polyurethane and air were calculated as η_p_ = 12.2% and η_a_ = 87.8%.

As a physical property, the true dielectric permittivity of monolithic polyurethane is independent of ambient air humidity. The permittivity spectrum ε_p_(f_n_) of monolithic polyurethane SikaBlock^®^-M960, measured at the lowest experimentally registered water vapor value m_w_ = 3.6 g/m^3^, was assumed to be sufficiently close to the true permittivity spectrum, corresponding to dry air m_w_ = 0.0 g/m^3^ and staying constant for all m_w_. On the contrary, the true dielectric permittivity of ambient air depends on humidity. At the lowest experimentally registered water vapor mass value m_wmin_ = 3.6 g/m^3^ ε_air_ = 1.000597 and at the highest m_wmax_ = 11.1 g/m^3^ ε_air_ = 1.000677 (Equation (33)), dependence of air permittivity on frequency was not taken into account.

The numerical calculations at the lowest and the highest m_w_ values are shown in Table 6. Even when the cellular structure of the entire sample of thickness h = 20 mm was filled with the most humid air (ε_air_ = 1.000677), the PU foams effective permittivity ε_f_(f_n_) differed from the effective permittivity at nearly dry air (ε_air_ = 1.000597) only in the fourth digit behind the decimal separator (0.01%). Thus, in the case of the open-cell bottom layer of thickness 0.20–0.25 mm (~100 X smaller than the height of the sample), humidity’s variations of air inside the sample alone may not cause the experimentally identified variations in the PU foams sample’s permittivity in the second digit behind the decimal separator (Figure 10 and Figure 13). The absorption of water by polymeric structural elements also has to be considered.

## 5. Conclusions

A year-long experimental investigation showed that the relative humidity of ambient atmosphere had the highest correlation with the measured values of dielectric permittivity of rigid PU foams and monolithic polyurethanes, while the temperature was much smaller and the correlation with pressure was insignificant.

A mathematical model of the effective dielectric permittivity suggested that the penetration alone of moist air into cells of PU foams cannot explain the experimentally detected variations in the measured values of permittivity. Other phenomena, namely the absorption of water by the polyurethane, have to be considered as well. Considering permittivity at 3 g/m^3^ as sufficiently close to the true permittivity value, the relative increase R(f_n_) in the permittivity value due to water absorption from ambient air is estimated numerically for rigid PU foams of densities 33–1280 kg/m^3^.

It can be concluded that at least the following two phenomena interfere with measuring of the true permittivity value of rigid PU foams: (1) the absorption of water from ambient air by a polar plastic – the monolithic polyurethane of the sample and (2) the warm-up drift of the COSA sensor system. The elaborated novel methodology, demonstrated in the paper for SikaBlock®-M150 rigid PU foams (as an example), permits to calculate the true permittivity spectrum for rigid PU foams of any density, based on the experimentally estimated dependence of dielectric permittivity on water vapor mass in air and warm-up drift of the COSA sensor system. The true permittivity spectrum is determined without any involvement of additional instrumentation (environmental chamber, desiccators, salt–water solutions, etc.).

It was shown that atmospheric moisture, even in the indoor laboratory environment, impacts the measured permittivity value of the preliminary unconditioned rigid PU foams and monolithic polyurethane. Impacts of the same origin can be expected in outdoor measurements. A water-proof coating of the sensitive parts of the capacitive sensor system might eliminate the impact of ambient air humidity inside the sensor on the dielectric permittivity’s measurements. More investigations would be necessary in that direction.

Although the quantitative results are valid for the given COSA sensor system with its topography of the electric field, the elaborated methodology may be applied to capacitive sensors of other configuration and dimensions of electrodes, independent of the particular technical solution, as well as in the investigation of the dielectric permittivity of other polar plastic foams.

## Figures and Tables

**Table 1:** The intervals of water vapor mass.

Interval Number i	Water Vapor Mass in Air m_w_; g/m^3^
1	3.0 ≤ m_w_ < 4.0
2	4.0 ≤ m_w_ < 5.0
3	5.0 ≤ m_w_ < 6.0
4	6.0 ≤ m_w_ < 7.0
5	7.0 ≤ m_w_ < 8.0
6	8.0 ≤ m_w_ < 9.0
7	9.0 ≤ m_w_ < 10.0
8	10.0 ≤ m_w_ < 11.0
9	11.0 ≤ m_w_ < 12.0

**Table 2:** Correlation between the permittivity and atmospheric parameters in the short series.

№	Atmospheric Parameter	Range of Values	Pairs of Variables	Coefficient of Determination R^2^
1	T; °C	14.4–25.2	“ε-T”	19% ≤ R^2^ ≤ 35%
2	p; hPa	980–1040	“ε-p”	5% ≤ R^2^ ≤ 9%
3	RH; %	21–60	“ε-RH”	65% ≤ R^2^ ≤ 94%

**Table 3:** Measurement uncertainties of permittivity of PU SikaBlock^®^-M150 foams (density of 144 kg/m^3^) (short series).

Frequency	Interval	1	2	3	4	5	6	7	8	9	All
	m_w_; kg/m^3^	3.0–4.0	4.0–5.0	5.0–6.0	6.0–7.0	7.0–8.0	8.0–9.0	9.0–10.0	10.0–11.0	11.0–12.0	3.1–11.1
	K_i_	16	30	14	7	14	32	10	10	1	134
f_1_ = 10 Hz	ε_3av_	1.259	1.265	1.269	1.278	1.278	1.282	1.286	1.289	1.293	1.274
	u(ε_3_)	0.005	0.005	0.005	0.005	0.005	0.003	0.004	0.003	-	0.011
	U95%	0.009	0.011	0.009	≥0.011	0.009	0.006	0.008	0.006	-	0.022
f_3_ = 40 Hz	ε_3av_	1.254	1.259	1.262	1.271	1.271	1.275	1.279	1.282	1.287	1.268
	u(ε_3_)	0.004	0.005	0.004	0.005	0.005	0.003	0.003	0.003	-	0.010
	U95%	0.009	0.010	0.009	≥0.011	0.009	0.006	0.007	0.006	-	0.020
f_5_ = 160 Hz	ε_3av_	1.249	1.254	1.256	1.264	1.265	1.268	1.272	1.274	1.281	1.262
	u(ε_3_)	0.004	0.004	0.004	0.005	0.005	0.003	0.003	0.003	-	0.009
	U95%	0.008	0.009	0.008	≥0.010	0.009	0.006	0.006	0.006	-	0.018
f_7_ = 640 Hz	ε_3av_	1.244	1.248	1.250	1.257	1.258	1.261	1.265	1.267	1.273	1.255
	u(ε_3_)	0.004	0.004	0.003	0.006	0.005	0.003	0.003	0.003	-	0.008
	U95%	0.007	0.008	0.007	≥0.011	0.009	0.005	0.006	0.006	-	0.017
f_9_ = 2.56 kHz	ε_3av_	1.239	1.243	1.244	1.250	1.250	1.253	1.257	1.259	1.265	1.248
	u(ε_3_)	0.003	0.004	0.003	0.005	0.004	0.003	0.003	0.003	-	0.007
	U95%	0.007	0.008	0.006	≥0.010	0.008	0.005	0.005	0.006	-	0.014
f_11_ = 10.24 kHz	ε_3av_	1.234	1.237	1.238	1.243	1.243	1.245	1.248	1.250	1.257	1.242
	u(ε_3_)	0.003	0.004	0.003	0.004	0.004	0.002	0.002	0.003	-	0.006
	U95%	0.006	0.007	0.005	≥0.009	0.008	0.004	0.005	0.005	-	0.012
f_13_ = 40.96 kHz	ε_3av_	1.229	1.232	1.231	1.236	1.236	1.237	1.239	1.242	1.247	1.235
	u(ε_3_)	0.003	0.004	0.002	0.003	0.003	0.002	0.002	0.002	-	0.005
	U95%	0.006	0.007	0.004	≥0.007	0.006	0.004	0.004	0.004	-	0.009
f_15_ = 163.84 kHz	ε_3av_	1.224	1.227	1.225	1.228	1.228	1.229	1.230	1.233	1.237	1.228
	u(ε_3_)	0.003	0.003	0.002	0.002	0.002	0.002	0.002	0.002	-	0.003
	U95%	0.006	0.007	0.004	≥0.005	0.005	0.003	0.004	0.003	-	0.007

**Table 4:** The statistical characteristics of atmospheric parameters in the 9 intervals of water vapour mass in air m_w_ (short series).

Interval Number	Statistical Characteristics	Temperature T; °C	Rel. Humidity RH; %	Pressure p; hPa	m_w_; g/m^3^	Interval Number	Temperature T; °C	Rel. Humid. RH; %	Pressure p; hPa	m_w_; g/m^3^
1	Average value	17.7	26	1017	3.7	6	21.5	49	1015	8.5
	Stand. deviation	0.8	3	13	0.3		1.1	3	9	0.3
	Lower value	16.1	20	991	3.1		19.3	43	997	7.9
	Upper value	19.3	31	1043	4.3		23.6	55	1034	9.1
2	Average value	17.3	32	1009	4.4	7	21.9	52	1016	9.4
	Stand. deviation	1.6	3	13	0.2		1.1	3	5	0.2
	Lower value	14.2	25	983	4.0		19.7	47	1006	9.0
	Upper value	20.4	39	1035	4.9		24.1	58	1026	9.8
3	Average value	18.1	37	1017	5.4	8	23.4	54	1017	10.5
	Stand. deviation	1.8	4	19	0.3		1.4	4	3	0.3
	Lower value	14.5	29	980	4.9		20.7	47	1011	9.9
	Upper value	21.7	46	1054	5.9		26.2	61	1023	11.1
4	Average value	18.0	45	1018	6.4	9	22.4	60	1016	11.1
	Stand. deviation	2.1	5	7	0.3		-	-	-	-
	Lower value	13.8	34	1005	5.9		-	-	-	-
	Upper value	22.1	56	1032	7.0		-	-	-	-
5	Average value	20.5	45	1012	7.5					
	Stand. deviation	1.2	3	6	0.3					
	Lower value	18.1	39	999	7.0					
	Upper value	22.9	52	1025	8.0					

**Table 5:** The characteristics of polyurethane materials.

№	Polyurethane Material	Density ρ; kg/m^3^	Volume Fraction of PU η_p_; %	ε(f_n_) at m_w_ = 3 g/m^3^			ε(f_n_) at m_w_ = 8 g/m^3^		
				f_3_	f_8_	f_14_	f_3_	f_8_	f_14_
1	PU foams	33	2.6	1.049	1.048	1.046	1.051	1.050	1.048
2		144	11.3	1.251	1.240	1.227	1.274	1.257	1.233
3		274	21.4	1.475	1.467	1.451	1.506	1.490	1.463
4		459	35.9	1.825	1.782	1.737	1.889	1.825	1.763
5		993	77.6	3.093	3.055	2.996	3.234	3.170	3.083
6	Monolithic PU	1 280	100.0	3.395	3.379	3.325	3.626	3.571	3.462
7		1 351	100.0	4.212	4.112	3.967	4.461	4.332	4.125

**Table 6:** The effective permittivity of open-cell PU SikaBlock^®^-M150 foams (density of 144 kg/m^3^).

Frequency f_n_; Hz	Permittivity of Polyurethane	Effective Permittivity ε_f_(f_n_)	
		m_wmin_	m_wmax_
f_1_ = 10 Hz	3.767	1.26342	1.26350
f_2_ = 20 Hz	3.757	1.26255	1.26263
f_3_ = 40 Hz	3.745	1.26144	1.26152
f_4_ = 80 Hz	3.732	1.26034	1.26042
f_5_ = 160 Hz	3.721	1.25938	1.25946
f_6_ = 320 Hz	3.710	1.25841	1.25848
f_7_ = 640 Hz	3.698	1.25738	1.25745
f_8_ = 1280 Hz	3.688	1.25651	1.25658
f_9_ = 2560 Hz	3.677	1.25551	1.25558
f_10_ = 5120 Hz	3.664	1.25439	1.25447
f_11_ = 10,240 Hz	3.651	1.25324	1.25331
f_12_ = 20,480 Hz	3.635	1.25185	1.25192
f_13_ = 40,960 Hz	3.615	1.25007	1.25014
f_14_ = 81,920 Hz	3.590	1.24790	1.24797
f_15_ = 163,840 Hz	3.560	1.24528	1.24535
f_16_ = 327,680 Hz	3.525	1.24215	1.24223

## Data Availability

Data are contained within this article.

## References

[1] Hilyard N.C. (1982). Mechanics of Cellular Plastics.

[2] Klempner D., Frisch K.C. (1991). Handbook of Polymeric Foams and Foam Technology.

[3] Gibson L.J., Ashby M.F. (1997). Cellular Solids: Structures and Properties.

[4] SikaBlock®-M150. Product Data Sheet. https://industry.sika.com/en/home/advanced-resins/model-and-mold-manufacturing/block-materials/design-and-stylingboards/sikablock-m150.html.

[5] SikaBlock®-M960. Product Data Sheet. https://industry.sika.com/en/home/advanced-resins/model-and-mold-manufacturing/block-materials/boards-for-toolingandfoundryapplications/sikablock-m960.html.

[6] Goods S.H., Neuschwanger C.L., Henderson C.C., Skala D.M. (1998). Mechanical properties of CRETE, a polyurethane foam. J. Appl. Polym. Sci..

[7] Beverte I., Shtrauss V., Kalpinsh A., Lomanovskis U., Cabulis U., Sevastyanova I., Gaidukovs S. (2020). Dielectric Permittivity of Rigid Rapeseed Oil Polyol Polyurethane Biofoams and Petrochemical Foams at Low Frequencies. J. Renew. Mater..

[8] Beverte I., Cabulis U., Gaidukovs S. (2021). Polytetrafluoroethylene Films in Rigid Polyurethane Foams’ Dielectric Permittivity Measurements with a One-Side Access Capacitive Sensor. Polymers.

[9] Kremer F., Schonhals A. (2003). Broadband Dielectric Spectroscopy.

[10] Matiss I. (1982). Capacitive Transducers for Non-Destructive Testing.

[11] Matiss I., Kalpinsh A., Shtrauss V., Lomanovskis U., Bernavs I. (1991). Capacitance Transducer for NDT of Dielectric Properties of Materials. USSR copyright certificate; Bulletin; 12. https://worldwide.espacenet.com/patent/search/family/021418434/publication/SU1638665A1?q=SU1638665A1%20.

[12] Hu X., Yang W. (2010). Planar capacitive sensors—Designs and applications. Sensor Rev..

[13] Kalpinsh A., Shtrauss V., Lomanovskis U. (2013). Capacitive Probe for Non-Destructive Testing of Dielectric Materials. Patent Office of Republic of Latvia; 9. https://worldwide.espacenet.com/patent/search/family/050153973/publication/LV14728B?q=LV14728B.

[14] Kalpinsh A., Shtrauss V., Lomanovskis U. (2019). Digital Emulation of Dielectric Relaxation Functions for Capacitive Sensors of Non-Destructive Dielectric Spectrometry. Comp. Meth. Exp. Meas..

[15] Chen T. (2012). Capacitive Sensors for Measuring Complex Permittivity of Planar and Cylindrical Structures. Ph.D. Thesis.

[16] Avramov-Zamurovic S., Lee R.D. (2009). A High-Stability Capacitance Sensor System and Its Evaluation. IEEE Trans. Instrum. Meas..

[17] Santo Zarnik M., Belavic D. (2012). An Experimental and Numerical Study of the Humidity Effect on the Stability of a Capacitive Ceramic Pressure Sensor. Radioengineering.

[18] Santo Zarnik M., Belavic D. (2012). The Effect of Humidity on the Stability of LTCC Pressure Sensors. Metrol. Meas. Syst..

[19] Santo Zarnik M., Belavic D., Macek S. (2010). The warm-up and offset stability of a low-pressure piezoresistive ceramic pressure sensor. Sens. Actuators A.

[20] Nassr A.A., Ahmed W.H., El-Dakhakhni W.W. (2008). Coplanar capacitance sensors for detecting water intrusion in composite structures. Meas. Sci. Technol..

[21] Gao X., Zhao Y., Ma H. (2018). Fringing electric field sensors for anti-attack at system-level protection. Sensors.

[22] Chang X.M., Dou Y., Zhuo D., Fan J.H. Research on sensor of ice layer thickness based on effect of fringe electric field. Proceedings of the 2012 International Conference on Computing, Measurement, Control and Sensor Network.

[23] The MAC Humidity/Moisture Handbook, p.20. www.macinstruments.com.

[24] Baxter L.K. (1997). Capacitive Sensors.

[25] Yeap P.I., Yuhana N.Y., Fariz S., Otoh M.Z. (2020). Temperature on the Mechanical, Thermal and Barrier Property of Polyurethane. IOP Conf. Ser. Mater. Sci. Eng..

[26] Yi Y., Sun Y., Wang L., Xiao P. (2021). Impacts of moisture absorption on electrical properties of rigid polyurethane foam for composite post insulator of UHVDC transmission line. Int. J. Electr. Power Energy Syst..

[27] Evaluation of Measurement Data—Guide to the Expression of Uncertainty in Measurement. JCGM 100:2008 GUM 1995 with Minor Corrections, 120p. https://www.bipm.org/utils/common/documents/jcgm/JCGM_100_2008_E.pdf.

[28] International Vocabulary of Metrology—Basic and General Concepts and Associated Terms (VIM), 3rd Edition 2008 Version with Minor Corrections. JCGM 200:2012. https://www.bipm.org/utils/common/documents/jcgm/JCGM_200_2012.pdf.

[29] Beverte I. (2014). Determination of Highly Porous Plastic Foams’ Structural Characteristics by Processing LM Images Data. J. Appl. Polym. Sci..

[30] Renz R. (1977). Zum Zuegigen und Zyklischen Verfomungsverhalten Polymerer Hartschaumstoffe. Ph.D. Thesis.

